# The effects of mild hypothermia on the electrode insertion trauma in a murine whole organ cochlea culture

**DOI:** 10.3389/fnins.2023.1112243

**Published:** 2023-04-13

**Authors:** Joachim Schmutzhard, Werner Bader, Timo Gottfried, Daniel Dejaco, Rudolf Glückert, Joszef Dudas, Annelies Schrott-Fischer

**Affiliations:** Department for Otorhinolaryngology, Head and Neck Surgery, Medical University of Innsbruck, Innsbruck, Austria

**Keywords:** hypothermia, whole organ cochlea culture, electrode insertion trauma, cochlear implant, sensorineural hearing loss, hearing rehabilitation

## Abstract

**Introduction:**

Local therapeutic hypothermia (32°C) has been linked experimentally to an otoprotective effect in the electrode insertion trauma. The pathomechanism of the electrode insertion trauma is connected to the activation of apoptosis and necrosis pathways, pro-inflammatory and fibrotic mechanisms. In a whole organ cochlea culture setting the effect of therapeutic hypothermia in an electrode insertion trauma model is evaluated.

**Material and Methods:**

The cochleae of C57Bl6/J mice (Charles River®, Freiburg, Germany) are cultured for 24 hours at 37°C and 32°C after inserting a fishing line through the round window simulating an insertion trauma. The resulting effect was evaluated for the apoptotic reaction – B-cell-Lymphoma-2-Associated-X-Protein (BAX), B-Cell-Lymphoma-2-Protein (BCL2) and Cleaved-Caspase-3 (CC3) –, the inflammatory response – Tumor-Necrosis-Factor-Alpha (TNFα), Interleukin-1-Beta (IL-1Imm) and Cyclooxygenase-2 (COX2) – and proliferation process – Transforming-Growth-Factor-Beta-1 (TGFβ1) – using immunohistochemistry and real-time PCR technique. A minimum of 12 cochlea per experiment were used.

**Results:**

A pro-apoptotic situation was observed in the normothermic group (BAX, CC3 ˃ Bcl2) whereas an anti-apoptotic constellation was found at 32°C culture conditions (BAX, CC3 < Bcl2). Furthermore the effect of the IT knowing to effect the pro-inflammatory cytokine (TNFα, Il1β) and enzyme (COX2) expression has been reproduced. This reaction was reversed with the application of therapeutic hypothermia resulting in significant lower pro-inflammatory cytokine (TNFα, Il1β) and enzyme (COX2) expression. TGFβ1 was increased by hypothermia.

**Discussion:**

Concluding a protective effect of hypothermia on the experimental electrode insertion trauma can be described by an anti-apoptotic and anti-inflammatory reaction.

## 1. Introduction

The otoprotective effect of hypothermia has been described by Henry et al. in the early 1980. In a rodent experiment utilizing whole body hypothermia a significant otoprotection was described in relation to noise induced hearing loss (Henry and Chole, 1984). Furthermore, the protection of the auditory function applying whole body hypothermia was examined in other otic insults, like cochlea ischemia or cochlea implantation (Watanabe et al., 2001; Balkany et al., 2005). In the meantime, the application of therapeutic hypothermia to the ear has been optimized to local application strategies. In a rodent animal experiment Smith et al. applied cold saline irrigation as cooling medium achieving local cochlea hypothermia with stable rectal body temperatures (Smith et al., 2007). The cold saline irrigation technique has been further evaluated lately as sufficient cooling strategy in human temporal bones achieving therapeutic hypothermia throughout the entire cochlea (Bader et al., 2020). Furthermore, alternative applications of localized cochlea hypothermia have been evaluated in various ototoxic setups. Spankovich et al. showed a reduced cisplatin-related hearing loss by cold water irrigation in guinea pigs (Spankovich et al., 2016). In an alternative rodent experiment, the otoprotective effect of hypothermia applied through a custom-made cooling device was evaluated in a cochlea implantation situation. In this study, cochlea implantation was performed in normothermic and hypothermic rodent cochlea. The hypothermic group showed significant hearing preservation in contrast to the normothermic animals. Furthermore, the hearing preservation could be linked to a preservation of the outer hair cells (Tamames et al., 2016). This *in vivo* experiment revealed the positive effect of hypothermia on the electrode insertion trauma and the subsequent loss of residual hearing caused during cochlear implant surgery.

The decline of residual hearing—caused by mechanical trauma (Eshraghi et al., 2003)—in cochlear implant surgery can be divided into early hearing loss and delayed hearing loss. Early hearing loss has been linked to the activation of apoptosis and necrosis pathways as well as the generation of reactive oxygen species and further free radicals and can be modulated by otoprotective agents (Eshraghi et al., 2007). The delayed hearing loss is argued to be the result of fibrotic proliferation of tissue within the cochlea (Jia et al., 2013). These mechanisms were mapped out by Bas et al. in detail 2012 utilizing an electrode insertion trauma in rodent whole organ cochlea cultures (Bas et al., 2012).

In the early response, an upregulation of the pro-inflammatory cytokines Tumor-Necrosis-Factor-Alpha (TNFα) and Interleukin-1-Beta (IL1β) as well as the pro-inflammatory enzymes Inducible Nitric-Oxide-Synthetase (iNOS) and Cyclooxygenase-2 (COX2) could be observed in the electrode insertion trauma group. Furthermore, an increased production of the total reactive oxygen species (ROS), an activation of Cleaved-Caspase-3 (CC3) and a Transforming-Growth-Factor-Beta-1 (TGFβ1) upregulation was observed in the electrode insertion trauma group. Based on these findings the authors described a two phased reaction in electrode insertion trauma consisting of an early inflammation and a later proliferation process (Bas et al., 2012).

The pathophysiological effect of therapeutic hypothermia in otic insults has not been fully examined at this point. Various potential mechanisms have been described. Nevertheless, a large knowledge gap with regard to the exact mechanisms exists (Spankovich and Walters, 2021). The question of the included pathophysiological mechanism of therapeutic hypothermia on stroked central nervous system tissue has been already examined further in detail. In stroke, a temperature-dependent reduction of the free radicals could be shown. Furthermore, the activation of pro-apoptotic proteins such as B-Cell-Lymphoma-2-Homologous-Antagonist/Killer (BAK) and B-Cell-Lymphoma-2Associated-X-protein (BAX), is downregulated, whereas the anti-apoptotic activation of (B-CellLymphoma-2 (BCL2) is amplified. Additionally, the expression of the pro-inflammatory cytokines TNFα and Il-1β is reduced (Lee et al., 2017). All these mechanisms have been shown to participate in the pathophysiology of the electrode insertion trauma described by Bas et al. (2012).

The presented paper aims to further evaluate the pathophysiologic effects of therapeutic hypothermia on the electrode insertion trauma in the previous established and utilized whole organ cochlea culture approach.

## 2. Materials and methods

Deeply anesthetized animals were euthanized by rapid cervical dislocation. Extraction of tissue from euthanized animals conforms with the Austrian Federal act on Experiments of Living Animals (Tierversuchsgesetz 2012—TVG 2012, §2) based on the EU Directive 2010/63/EU. Thus, ethical review and approval was not required for the present animal study. No human studies are presented in this manuscript. No potentially identifiable human images or data are presented in this study.

### 2.1. Study design

C57-Black-6/J (C57Bl6/J) mice (Charles River®, Freiburg, Germany) were used for this *in vitro* experiment. 10 day old mice were scarified and the inner ears prepared within 3 min. Afterward, a probe was inserted into the prior opened round window though the basal turn of the cochlea imitating an electrode insertion trauma as published by Bas et al. (2012). Additionally, the experiment was performed on the equal number of cochlea without insertion trauma. Afterward, the traumatized cochlea were cultured for 24 h as described below. The cochleae were divided into a normothermic group—culture conditions at 37°C—and a therapeutic hypothermic group—culture conditions at 32°C. After a 24 h cultivation period, the cochlea were harvested and further processed with immunohistochemistry or ribonucleic acid (RNA) isolation or polymerase chain reaction (PCR) technique. Experiments were repeated 3 times for each treatment at 37°C and 32°C with 3–4 cochleae.

The culture integrity was confirmed with light microscopy, using 4′,6-diamidino-2-phenylindole (DAPI) and Phalloidin staining as well as immunohistochemistry for Myosin VIIa and β-III-Tubulin. The apoptotic pathway was examined with immunohistochemistry for BAX, BCL2, and CC3. The inflammatory response was evaluated with quantitative polymerase chain reaction (qPCR) analysis for TNFα, IL1β, and COX2. The later proliferation process was examined by TGFβ1 qPCR.

### 2.2. Whole organ cochlea culture

#### 2.2.1. Cochlea dissection and preparation

Ten day old C57BL6/J mice (Charles River®, Freiburg, Germany) were used for the Rotary Culture experiments. Animals were sacrificed in deep anesthesia—intraperitoneal ketamine hydrochloride (Graeub®, Senden-Bösensell, Germany) (67.5 mg/kg body weight), xylazine hydrochloride (Bayer®, Leverkusen, Germany) (5.4 mg/kg body weight) and atropine sulfate (Nycomed®, Linz, Austria) (0.085 mg/kg)—by cervical dislocation and decapitated. Afterward, the bulla auditiva was prepared and the cochleae were identified. Inner ears were then transferred into a Petri dish containing cooled Hank’s balanced Salt Solution (HBSS) media (Gibco™, Thermo Fisher Scientific®, Germany)—without magnesium chloride (MgCl^2^)—or Neurobasal media (Gibco™, Thermo Fisher Scientific®, Germany). HBSS media was especially used in the dissecting process in the Petri dish. After dissecting the cochlea, it was transferred in a 10 ml disposable Rotary vessel (SYNTHECON INC. ®, Houston, Texas), filled with cooled Neurobasal media. Cochleae were opened using a 45° surgical needle at apical and basal turn. At the oval window, the cochleae were rinsed gently throughout over the whole cochlea. Only, the first dissected cochleae were further processed for the rotary culture to limit preparation time and ischemic effects to the cochlea culture. Then the selected cochleae were put in a 24 well-plate containing HBSS media—without MgCl^2^—and positioned on a lab shaker. The culturing process was performed on a non-insertion trauma cochlea group (NIT) and an insertion trauma group (IT). The electrode insertion trauma was set using a probe, simulating a cochlea implant.

Therefore, a 0.1 mm in diameter nylon thread (Waterqueen®, Bielsko-Biala, Poland) was cut into short pieces of approximately 3 cm. With a surgical microscope, the probe was inserted through the round window in the Scala tympani and left in place. Insertion trauma cochleae were then cultured as described below.

#### 2.2.2. Rotary culture setup

The Rotary Cell Culture System (RCCS) (Hahn et al., 2008) (SYNTHECON INC., Houston, Texas) with four rotating positions was used for the culture experiments. In experimental setups, RCCS disposable vessels (SYNTHECON INC., Houston, Texas) of 10 ml volume were used. For maintenance of simulated body temperature at 37°C and mild hypothermia at 32°C, a CO_2_ Water Jacketed Incubator (Forma Scientific™, Thermo Fisher Scientific®, Marietta, Ohio) was used simulating stable temperature gradients. The temperature of the incubator was constantly monitored with an additional internal temperature probe. The composition of the Rotary Culture Media is listed in the supplementary data (Supplementary Table A). Additionally, neurotrophic factors in a concentration of 10 ng/ml Brain-Derived-Neurotrophic-Factor (BDNF) (PeproTech® EC, ltd., London, United Kingdom, cat. 450-02-10UG, lot. 071961 H2420) combined with 5 ng/ml Neurotrophin-3 (NT3) (PeproTech® EC, ltd., London, United Kingdom, cat. 450–03-10UG, lot. 031962 G2220) were added to improve neuron-, outer hair cell (OHC), and inner hair cell (IHC) survival. The RCCS disposable vessels (10 ml) were gently filled with culture medium (detailed in Supplementary Table A). This procedure was performed under the laminar flow to prevent further contamination of the culturing media. If both valves were opened, the vessel could be easily filled without any air bubbles. Afterward, the vessels were mounted on the rotating bioreactor device by turning them clockwise. A rotation velocity between 40 and 50 rpm was needed to prevent the mouse cochleae from colliding with the wall of the vessel. The culture conditions were maintained for 24 h.

### 2.3. Cochlea preparation

After an incubation time of 24 h at the chosen temperature, the cochleae were transferred into 2.00% (w/v) paraformaldehyde (PFA) in 0.5 fold diluted neurobasal medium at pH 7.4 for fixation for 24 h. Afterward, the cultivated cochleae were washed 5 times for 5 min each, in phosphate-buffered saline (PBS; 1x) and decalcified in 20% Ethylenediaminetetraacetic acid (EDTA; pH 7.4) for 220 min in the microwave at 800 Watt. The cryo-embedding was performed according to a standard procedure (Coleman et al., 2009).

Cryo-sectioning was performed with a Leica CM 3050 system (Leica Microsystems®, Mannheim, Germany). Sections were cut at 5 μm slices and transferred to microscopic slides. Afterwards the slides were stored at – 20°C for further Immunohistochemisty.

Four 10 day old C57BL6/J (Charles River®, Freiburg, Germany) cochleae were fixed with Karnovsky (Spoendlin and Schrott, 1987; Johnson Chacko et al., 2019) fixative and further processed according to a standard semi thin section protocol with toluidine blue staining to visualize the structural preservation.

### 2.4. Staining procedures

#### 2.4.1. DAPI-staining

For DAPI (Life Technologies™, Invitrogen®, Inc., Darmstadt, Germany), fluorescence staining use a concentration of 1:20000 (5 μl in 100 ml) in Ventana Reaction Buffer (Roche Diagnostics®, Rotkreuz, Switzerland). Slides were exposed in this solution for 10 min and washed afterwards 5 times for 5 min each using PBS (1×).

#### 2.4.2. Phalloidin staining

Subsequently after the last washing step of DAPI (Life Technologies™, Invitrogen®, Inc., Darmstadt, Germany) staining, a Phalloidin fluorescein-isothiocyanate (FITC) (Sigma-Aldrich®, Vienna, Austria) staining was performed. Phalloidin FITC (Sigma-Aldrich®, Vienna, Austria) was used in a concentration of 1:20 [5 μl Phalloidin and 95 μl PBS (1x)] and directly pipetted on the slides under wet conditions. Microscopic slides were exposed in dark conditions for 40 min and washed 5 times with PBS (1×) 10 min each. To diminish possible background fluorescence, a further washing steps using PBS (1×) was applied. Afterwards, slides were mounted with VectaShield (VECTOR Laboratories® Inc., Burlingame, CA, United States), sealed with nail polish and stored then at 4°C in the fridge prior elaborating on a TissueFaxs system (TissueGnostics®, Vienna, Austria).

#### 2.4.3. Immunohistochemistry (myosin VIIa, β-III-tubulin, BAX, BCL2, CC3)

The cryo-section were washed for 10 min. in Ventana Reaction Buffer (Roche Diagnostics®, Rotkreuz, Switzerland) and fluorescence staining was performed using a Ventana discovery staining system (Roche Diagnostics®, Rotkreuz, Switzerland) using the standard protocol for cryo sections (frozen 402). To show the preservation of the nervous tissue, a concentration of 1:100 for beta-3Tubulin (Abcam® plc., Cambridge, United Kingdom, cat.-nr. ab52623) antibody was selected. The condition of IHC as well as OHC were illustrated with a Myosin-VIIa primary antibody (Proteus Biosciences® Inc., Ramona, CA, United States, cat.-nr. 25–6,790).

The apoptotic activity in the tissue was visualized with a primary antibody for CC3 at a concentration of 1:400 (Cell Signaling®, Leiden, Netherlands, lot. 47/9661S), BAX 1:10 (Santa Cruz Biotechnology® Inc., Dallas, Texas, United States, lot. B2912) and as antagonist BCL2 1:50 (Santa Cruz Biotechnology® Inc., Dallas, Texas, United States, lot. C0805 and lot. C0207) was utilized. As secondary antibody Alexa Rabbit 594 (Life Technologies™, InVitrogen® Inc., Darmstadt, Germany, ref. A21207, lot. 2,313,074) was selected in a concentration of 1:200.

#### 2.4.4. Data analysis

Antibody fluorescence stainings are further examined and photographed in a blinded manner with a Zeiss Axioplan (Carl Zeiss® Inc., Oberkochen, Germany) adjusted with a TissueFaxs device (TissueGnostics®, Vienna, Austria). For fluorescence imaging on TissueFaxs, a preview objective 2,5x (air) and for further acquisition an objective 40x (air) was used. In the acquisition mode Dapi filter was used as master channel. For Phalloidin FITC, the green filter (44FI) was used and the red filter (71HC) was used to visualize the marker. It is possible to acquire 8 slides on TissueFaxs microscope. Per slide, generally 3–4 mid-modiolar sections were defined as ROI (region of interest) and further evaluated.

Tissue Faxs files were further statistically examined in TissueQuest (TissueGnostics®, Vienna, Austria). Selected for quantitative evaluation in TissueQuest the menu item “Nuclear Segmentation.” After creating a new project file adjust to 44FI for Phalloidin as mastercanal, because our special focus were on Ganglia cells that are well stained with phalloidin. Following settings in the program are adjusted (Nuclei size = [25], area size smaller than 40 μm^2^ and larger than 300 μm^2^, max. Combined area = [4500], max. Involved compactness = [0.9], use of identified cell mask = [outside & inside]). These default settings were used for creating a template file for further analysis. Appropriate definition based using the Isotype control of the limit value (cutoff definition) (set and define the upper 10%, usually between 400 and 600 intensity in arbitrary units). Change to an antibody staining section and carry out these present parameters for further analysis. Subsequently change to the intensity graphics (scatter plot and histogram) for channel 71HC and convert the X-axis (horizontal axis) values to logrhythmic scale. Afterward, a statistical table in TissueQuest appears, showing the percentage of Marker positive as well as Marker negative Ganglia cells and intensity values. Statistical values like percentage and mean intensity were noted for each ROI (region of interest) for the 71HC fluorescence canal (red) in an Excel file. Per each immuno experiment for Bax, Bcl2 and CleavCas3, three replicates were obtained and the result noted in the Excel-file.

### 2.5. RNA isolation and real-time PCR for gene expression investigation of Tnfα, Il-1β, Cox2 and Tgf1β

Further inflammatory response genes, like Tgf1β, Tnfα, Il1β, and Cox2, were selected and their protein coding sequence was searched in National Center for Biotechnology Information (NCBI) database for species *Mus musculus*. Forward and reverse primers were designed and tested on possible self-annealing on several online tools like Primer3web (version 4.1.0), NCBI blast and the primer design tool by Thermo Fisher Scientific. The primers designed were produced by InVitrogen (Life Technologies™, InVitrogen® Inc., Darmstadt, Germany). For further RNA-analysis, different sample groups were set up; (1) “insertion trauma” (IT) at 37°C, (2) IT at 32° C, (3) “without insertion trauma” (NIT) at 37°C and 4) NIT at 32°C. As 5) “control group” (control), not treated 10 day old mouse cochleae of C57BL6/J (Charles River®, Freiburg, Germany) were collected. Three mouse cochleae were collected for each RCCS treatment and stored at −80°C in RNA later (Life Technologies™, InVitrogen® Inc., Darmstadt, Germany) in Eppendorf tubes. For each group this was repeated three times.

A standard RNA isolation was performed by the standard Trizol RNA isolation protocol (REF: 5596018) (Ambion, Life Technologies™, InVitrogen® Inc., Darmstadt, Germany). The RNA concentration was measured with an Eppendorf Biophotometer plus (Eppendorf®, Hamburg, Germany) and cDNA was prepared by reverse transcription using the protocol Superscript IV VILO (Life Technologies™, InVitrogen® Inc., Darmstadt, Germany) following the suggestions of the manufacturer. The forward and reverse primers used are listed in the supplementary material (Supplementary Table B).

For real-time quantitative polymerase chain reaction (rt-PCR) the Sensifast Sybr & Fluorescein kit (Bioline®, Lab consulting, Vienna, Austria) was used containing Reaction Buffer, DNA Polymerase, deoxynucleotide triphosphates (dNTPs), 6 mM MgCl^2^, Fluorescein and SybrGreen; as well as stabilizers. The components for a mastermix (Supplementary Table C) and the data for one rt-PCR cycle (Supplementary Table D) are detailed in the supplementary data. For qPCR a Bio-Rad MiIQ™ Single Color Real-Time PCR Detection System (Bio-Rad® ltd., Hercules, CA, United States).

#### 2.5.1. Statistical analysis for Bax, Bcl2 and Cc3

A dataset for Bax, Bcl2 and Cc3 was obtained in the statistical software GraphPad Prism 9.3.0.463 (GraphPad Software©, San Diego, CA, United States). To find out, whether the data obtained is following a Gaussian distribution or not, histograms in GraphPad were obtained to decide further statistical testing. For not normal distributed data, it was decided to use a two tailed Mann–Whitney test to compare two columns of different lengths. The percentage of number of “Marker”-positive cells counted in TissueQuest for Bax, Bcl2, and Cc3 divided by the number of Ganglion cells to compare median for 37°C and 32°C for NIT and IT for Bax, Bcl2, and Cc3. The relative frequency was afterwards visualized for each marker and each treatment in box plots indicating also the calculated significance in the Mann–Whitney test.

#### 2.5.2. Statistical analysis for quantitative PCR (qPCR)

After finishing qPCR, raw data were exported as comma-separated-values (CSV) files for Tgf1β, Cox2, Tnfα and Il1β in Excel and converted into common Excel format. Cycle-threshold values (CtValues) were summarized in treatment groups and possible missing data substituted by mean values. Mean value of replicates was calculated in Excel table and each experimental run was repeated three times. After sorting the data, Fold Expression Change for each pro-inflammatory marker was calculated and based on three repetitions of the same treatment group the mean fold expression as “mean fold units” (MFU) was calculated and summarized in Excel (Livak and Schmittgen, 2001). For each treatment, Fold Expression Change for the four groups (1) IT at 37° C, (2) IT at 32° C, (3) NIT at 37°C, (4) NIT at 32°C and (5) control were transferred in a GraphPad table. To test, whether the data is Gaussian distributed a normality-test was performed in GraphPad. For not normal distributed data to compare 37°C and 32°C data sets the Wilcoxon-Test was used.

## 3. Results

### 3.1. Structural preservation

#### 3.1.1. Semithin sectioning

**Figure 1:**
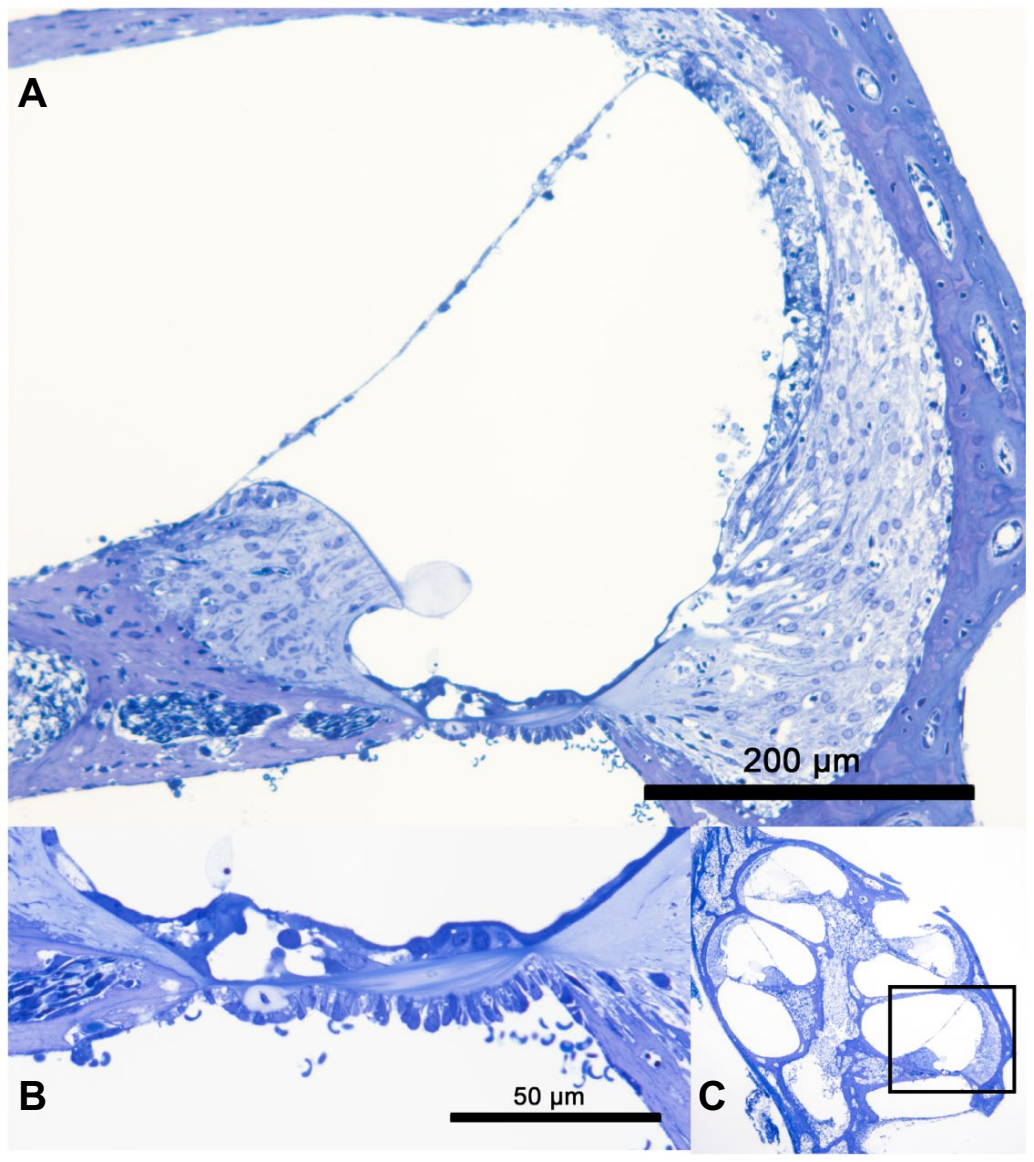
Panels **(A–C)** show a semi-thin tolluidin blue stained cochlea after 24 h in whole organ cochlea culture at 37°C. In panel **(A)** the cochlea duct of the basal turn is visualized. A good structural preservation of the organ of Corti, the stria vascularis, the spiral ganglion neuron and the spiral ligament can be observed. The high resolution of the organ of Corti is presented in panel **(B)**. The structural integrity of the OHC and IHC is shown. The overview of the whole organ of Corti with the marked shown cochlea duct is seen in panel **(C)**.

The structural preservation of the culture was verified with four random samples of cultured cochlea. The semi-thin toluidine blue stained section of a cochlea after 24 h in whole organ cochlea culture conditions is visualized in Figure 1. A highly satisfying structural preservation can be posted.

#### 3.1.2. Structural preservation with myosin VIIa and β-III-tubulin immunohistochemistry

The quality and preservation of the cultured murine cochlea was further examined by immunolabeling for Myosin VIIa and β-III-Tubulin. The Myosin VIIa antibody reacts with the actin filaments of the IHC and OHC proofing a hair cell survival. β-III-Tubulin binds to the neurofilaments of the cochlea visualizing an intact neuronal structures (Renauld et al., 2015).

Immunoreactivity was tested at 37°C and 32°C. A total of 12 cochlea (4 cochlea per culture with 3 culture repetitions) per temperature level were included for both reagents.

**Figure 2:**
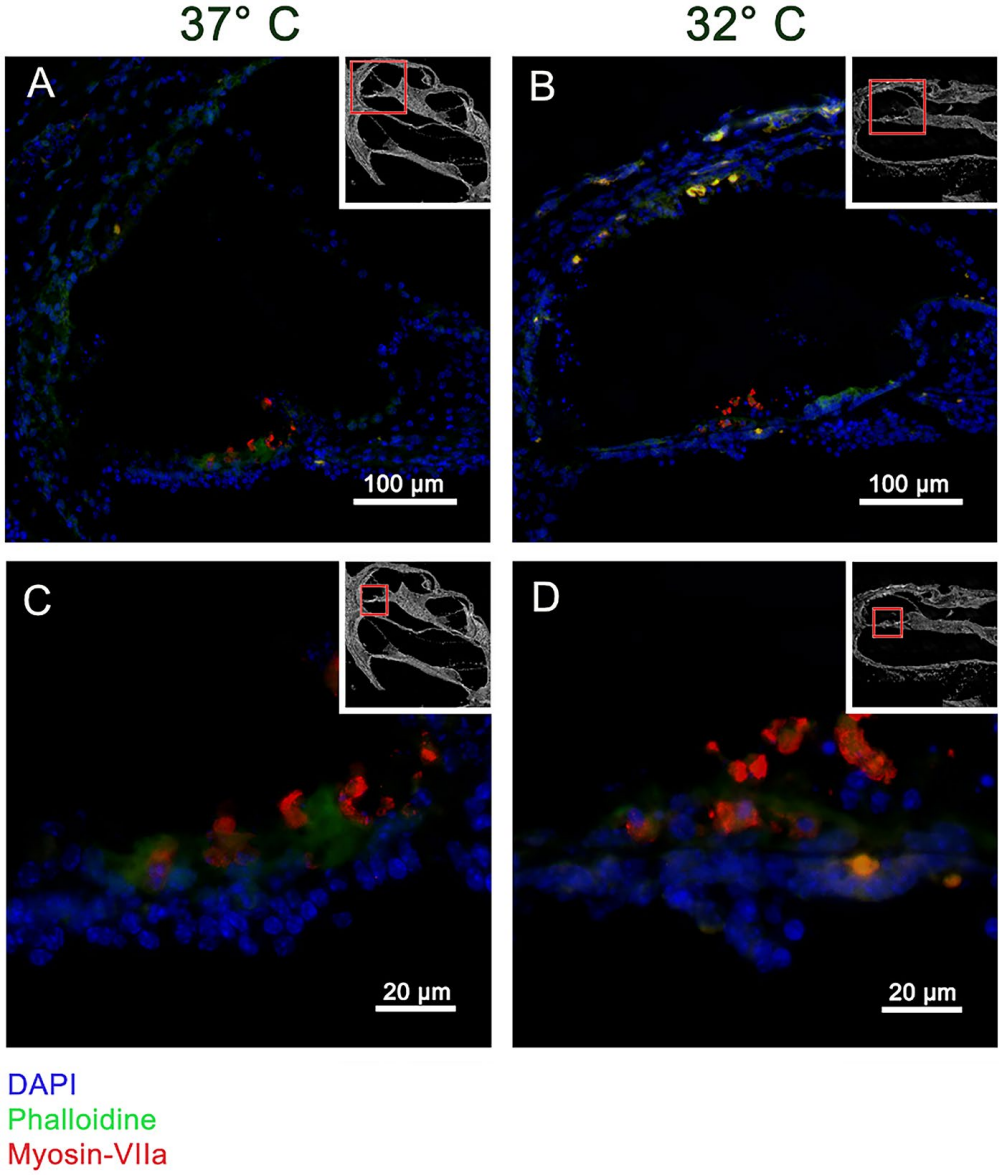
Panels **(A–D)** visualizes the immunohistochemistry for Myosin VIIa in the included murine whole organ cochlea culture samples. The inserts in each subset indicate an overview of the shown cochlea with a red square marking the position of the higher magnification. Panels **(A,C)** represent the results at 37°C culture temperature, whereas panels **(B,D)** stand for the 32°culture. The blue channel shows the DAPI staining, green the Phalloidin staining and red shows the Myosin VIIa reaction. The overview of the cochlea duct in panels **(A,B)** shows the selective coloring of the organ of Corti. The higher magnifications in panels **(C,D)** show the specific Myosin VIIa labeling of the IHC and OHC.

**Figure 3:**
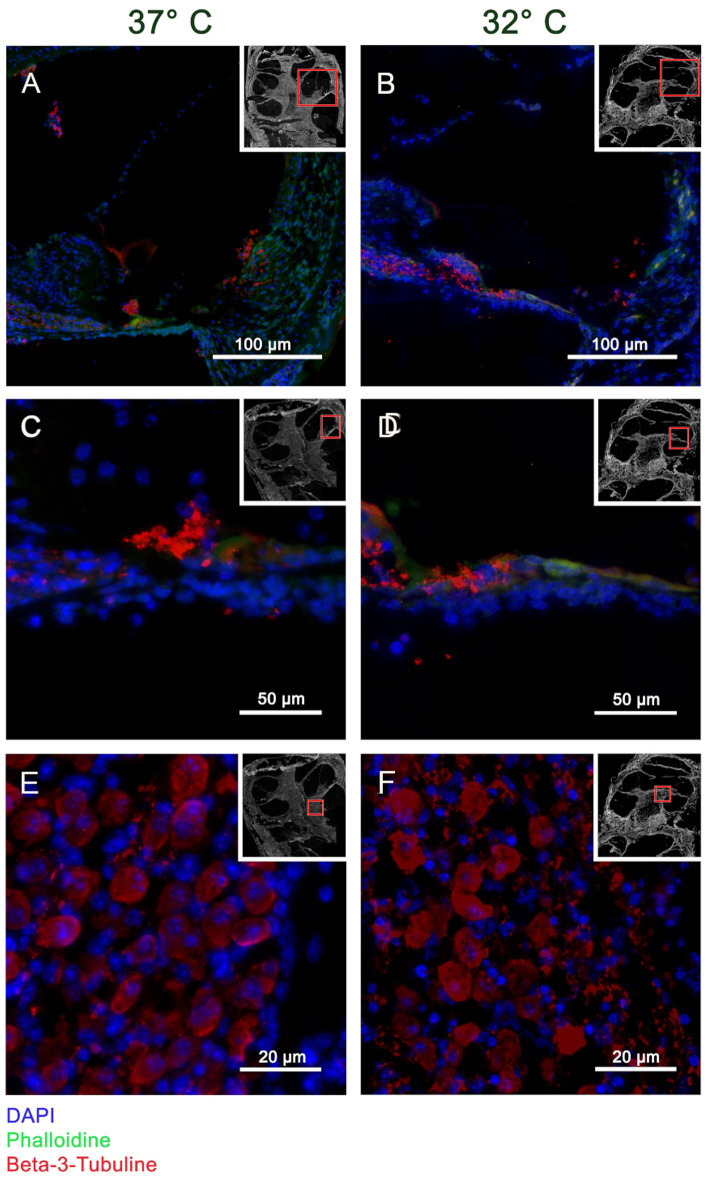
Panels **(A–F)** visualizes the immunohistochemistry for β-III-Tubulin in the included murine whole organ cochlea culture samples. The inserts in each subset indicate an overview of the shown cochlea with a red square marking the position of the higher magnification. Panels **(A,C,E)** represent the results at 37°C culture temperature, whereas panels **(B,D,F)** stand for the 32°culture. The blue channel shows the DAPI staining, green the Phalloidin staining and red shows the β-III-Tubulin reaction. The crossection of the cochlea duct in panels **(A,B)** shows a specific reaction of β-III-Tubulin in the neuronal fibers heading from organ of Corti to the spiral Ganglion. This observation is confirmed by the higher magnification of the medial basilar membrane in panels **(C,D)**. Panels **(E,F)** are high magnifications of the spiral ganglion proofing a specific labeling of the ganglia cells.

The Myosin VII a results are shown in Figures 2A–D. A selective staining within the organ of Corti can be posted at both temperature conditions suggesting stronger results at 32°C. Figure 3 shows the staining for β-III-Tubulin. A specific reactivity of the neuronal structures is shown in Figures 3A–F.

#### 3.1.3. Immunohistochemistry for the apoptotic markers—BAX, CC3, and BCL2

The apoptotic activity was examined with immunohistochemistry for the pro-apoptotic markers BAX and cleaved Caspase 3. The anti-apoptotic effect was visualized with labeling for Bcl2. The reactivity was tested in at 37°C and 32°C in a NIT and an IT group. A total of 12 cochlea (4 cochlea per culture with 3 culture repetitions) per temperature level with an applied electrode insertion trauma as described above were included for all reagents.

#### 3.1.4. BAX immunohistochemistry

**Figure 4:**
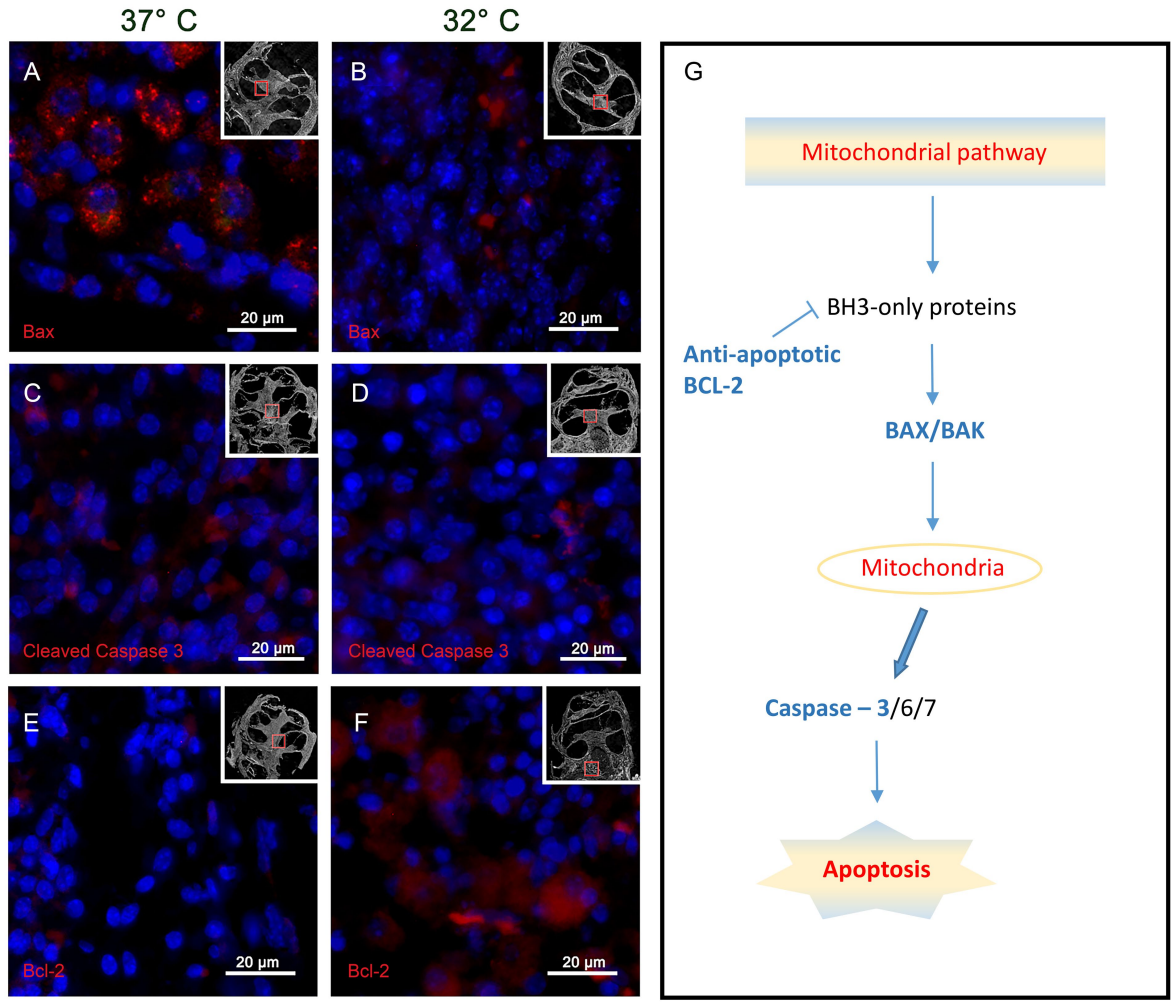
Shows the immunohistochemistry for the tested pro-apoptotic BAX, CC3 and the anti-apoptotic Bcl 2. The presented slides origin from the basal turn of the IT group at 37°C and 32°C. Panels **(A,B)** represents the results for BAX with a strong specific staining at 37°C **(A)** and a weak reactivity at 32°C **(B)**. The CC3 results are visualized in panels **(C,D)**. Again, a stronger reactivity can be posted at 37°C whereas a weaker coloring is noted at 32°C. The anti-apoptotic marker Bcl 2 is demonstrated by the inserts panels **(E,F)**. at 37°C almost no Bcl 2 labeling can be found **(E)**, whereas strong reactivity is found in the 32°C group(f). Panel **(G)** summarizes roughly the mitochondrial apoptosis cascades marking the tested proteins in blue. The small inserts in panels **(A–F)** show the region where the picture was taken in relation to the whole cochlea.

The results for the BAX immunohistochemistry in the IT group show higher reactivity in the 37° group, whereas the 32° group labeling is weaker. This observation can be observed best at the level of the spiral ganglion cells as shown in Figures 4A,B. A specific reaction in the soma of the ganglion cells can be shown in the 37°C group (Figure 4A), whereas almost no reaction is noted in the 32°C group (Figure 4).

#### 3.1.5. CC3 immunohistochemistry

The CC3 results in the IT group react accordingly to the above described BAX reactivity with more intensive staining in the 37° group and less reactivity in the 32°. Again, specific CC3 reactivity is shown in the soma of the spiral ganglion cells of the 37°C group as shown in Figure 4C. Clearly, less reaction is found in the 32°C group (Figure 4D).

#### 3.1.6. BCL2 immunohistochemistry

The anti-apoptotic activity is tested with immunoreactivity for BCL2 in the IT group as shown in Figures 4E,F. In the overview of the cochlea duct, more intensive staining is shown in the 32°C culture. This observation is further underlined in the higher magnifications of the spiral ganglion cells. At 37°C, only weak reactivity can be found (Figure 4E) whereas strong labeling can be posted at 32° (Figure 4F).

#### 3.1.7. Quantitative evaluation of immunohistochemistry for BAX, CC3, and BCL2

**Figure 5:**
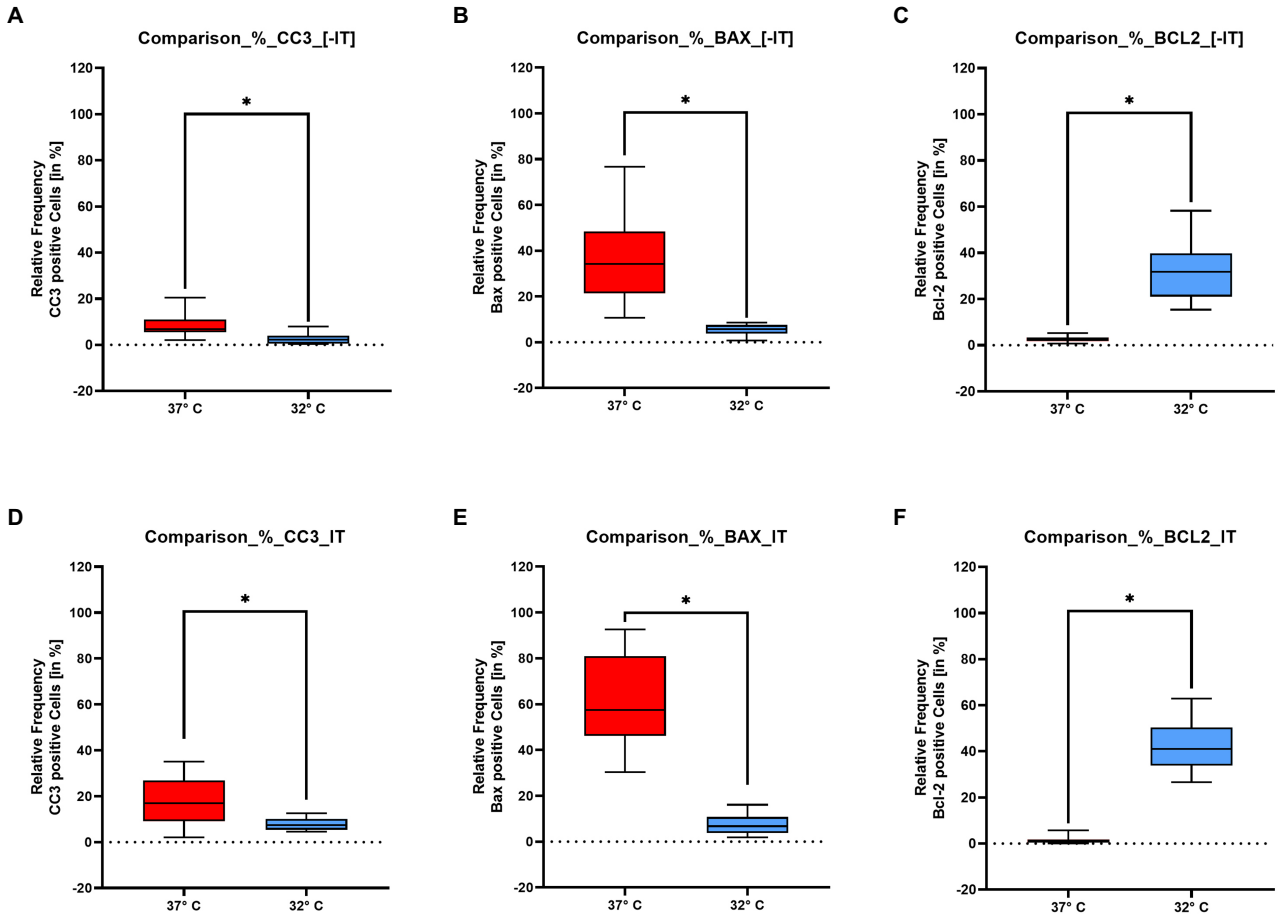
Show the quantitative results for the pro – apoptotic markers – BAX, Cleaved Caspase 3 – and the anti-apoptotic marker BCL 2 evaluated in the spiral ganglion for the NIT group panels **(A–C)** and the IT group panels **(D–F)**. The x-axis represents the two temperature groups at 37°C and 32°C. The y-axis stands for the positive percentage of ganglion cells in relation to the total number of ganglion cells. The results for *Cleaved Caspase 3* are shown in insert panels **(A,D)**. Both figures show a significant higher marking in the 37°. With a mean average of the NIT group is at 6.87% compared to 16.9% in the IT group. The *BAX* results are shown in panels **(B,E)**. The pro apoptotic BAX reacts significantly higher in the 37° collective. The NIT group with a mean percentage of positive cells of 34.2% is clearly lower as the IT collective with 57.4%. The expression pattern changes in the anti – apoptotic BCL 2 labeling shown in panels **(C,F)**. In the *BCL 2* series the results are higher in the 32° run. In the NIT situation the mean of the positive cells is at 31.8%, compared to 41% in the NIT group. The asterisks indicate a significant difference listed above for the Mann–Whitney-U test.

The above described observations are further underlined by the quantitative analysis, which has been performed for the NIT and the IT group. The quantitative analysis was performed on all 3 repetitions containing each 4 cochlea. So multiple mid-modiolar slices from 12 cochlea were included. The quantitative analysis was focused on the spiral ganglion cells of in the basal turn as shown in the inserts in Figures 4A–F. Significances were tested with the Mann–Whitney-U test. Comparing the positive spiral ganglion cells with the above presented tissue quest approach, a significant higher percentage of positive cells can be posted in the 37°C group for the pro-apoptotic markers BAX (IT *p* < 0.0001/ NIT *p* < 0.0001) and CC3 (IT *p* < 0.0001/NIT *p* = 0.0001) as shown in Figures 5A,B,D,E. The overall values were higher in the IT group than in the NIT group as summarized in Table 1. In contrast, the anti-apoptotic Bcl 2 was significant higher in the 32° group (IT *p* < 0.0001/NIT *p* < 0.0001) as demonstrated in Figures 5C,F. The overall values were higher in the IT group than in the NIT group as presented in Table 1.

**Table 1:** Descriptive results of the quantitative evaluation of the immunohistochemistry.

	Insertion trauma (IT)						Non insertion trauma (NIT)					
	Bax		CC3		Bcl2		BAX		CC3		Bcl2	
Temp.	37°C	32°C	37°C	32°C	37°C	32°C	37°C	32°C	37°C	32°C	37°C	32°C
Max.	92.56	16.06	35.07	12.50	5.73	57.50	62.98	8.51	20.3	7.91	5.26	58.21
Min.	30.23	1.77	2.10	4.50	0.00	26.54	10.76	0.65	2.14	0.38	0.77	15.39
Mean	57.39	6.71	16.96	7.43	0.83	41.06	34.21	5.70	6.87	2.16	2.47	31.86

### 3.2. Real-time PCR for gene expression of Il1β, Tnfα, Cox2 and Tgf1β

To test the data on statistical distribution, a Normality and Lognormality test in GraphPad Prism 9.3.0 was performed. The data of all four markers, Il1β, Tgf1β, Tnfα, and Cox2, are not normal distributed.

#### 3.2.1. Interleukin-1-beta (Il1ß)

**Figure 6:**
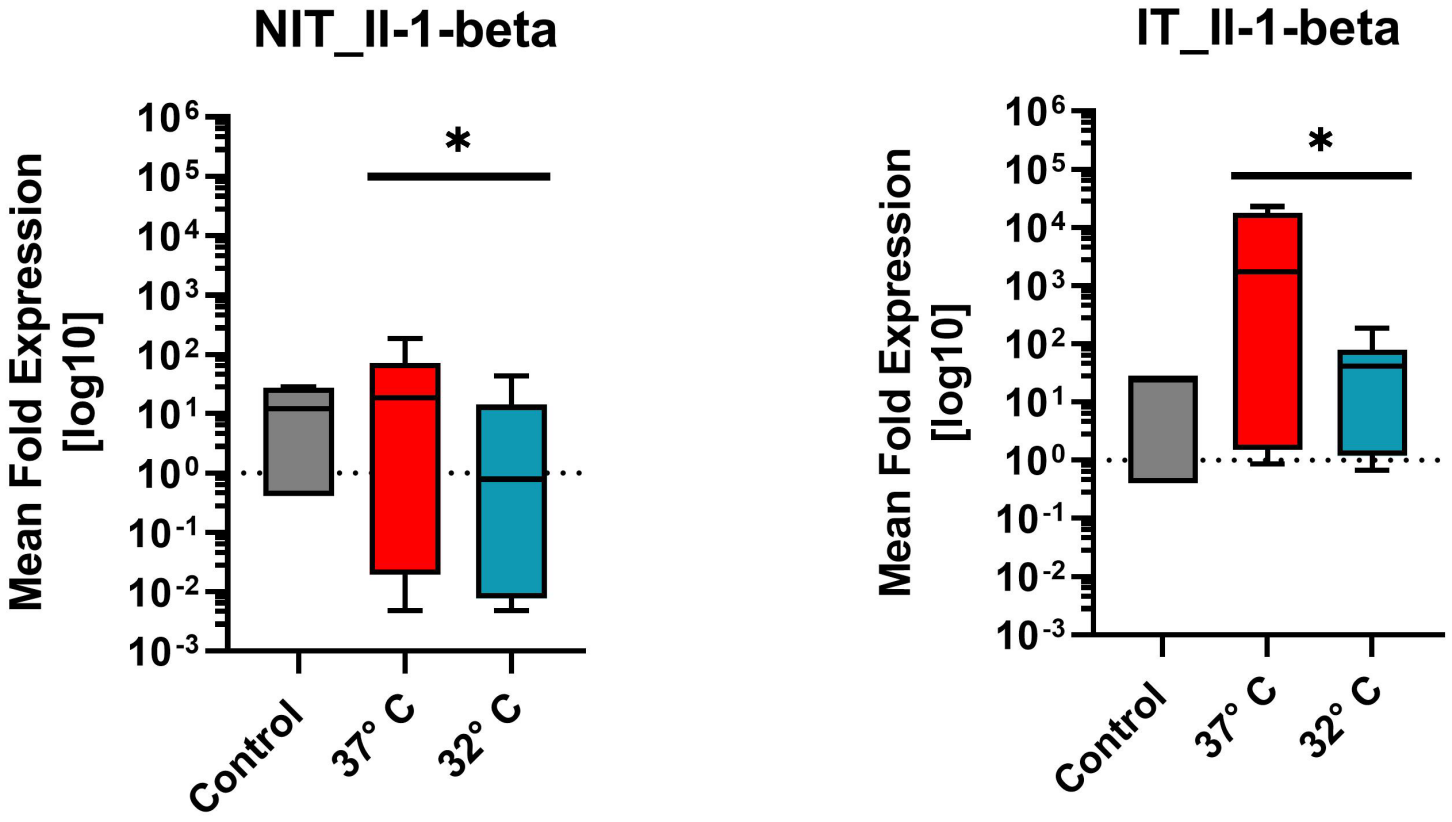
Comparison of mean expression fold changes of pro-inflammatory cytokine Il1beta for NIT versus IT. Control and two different temperature datasets at 3°C and 32°C were used to find out differences in the expression; every data column represents the mean of four replicates (*n* = 4) and three repetitions (*n* = 3). Comparing 3°C and 32°C data, a One-Sample-Wilcoxon-Test was performed for further statistical evaluation. A significance was calculated in NIT-treatment (value of *p* = 0.0005*) and in IT-treatment value of *p* was at 0.0039 (value of *p* = 0.0039*).

Il1ß belongs to the pro-inflammatory cytokine group. Figure 6 illustrates the mean fold expression of NIT versus IT. Control and two different temperature datasets at 37°C and at 32°C were used to find out differences in the activation. For further statistical evaluation a One-Sample-Wilcoxon-Test was performed. Comparing 37°C and 32°C in NIT, the median of MFU was at around 10 in the control group and at 37°C versus 32°C, the median level was located at around one. In IT, median level was also 10 in the control, for 37°C at around 10^3^ and for 32°C at 10^2^. A 10-fold reduction at 32°C compared with 37°C was present in both cases. According to the high median ± SEM for both treatments, showed a significance in the On-Sample-Wilcoxon-Test (value of *p* = 0.0005) for NIT and for IT (*p* = 0.0039) (Figure 6).

**Figure 7:**
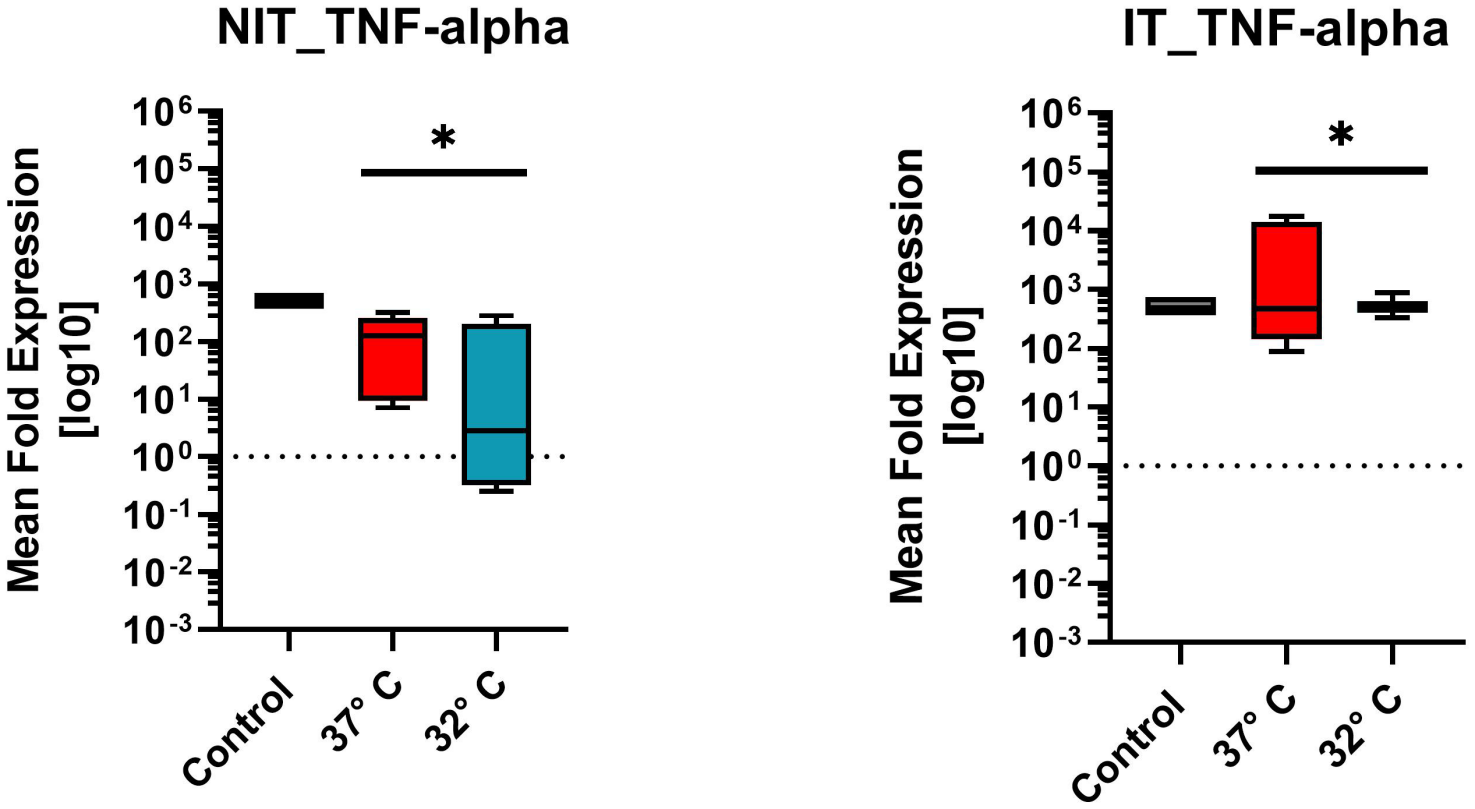
Comparison of mean fold expression of pro-inflammatory cytokine TNFα for NIT treatment as well as IT. Control and two different temperature datasets at 37°C and 3°C were used to find out differences in the expression; every data box represents the mean of four replicates (*n* = 4) and three repetitions (*n* = 3). The highest levels could be posted for the 37°C IT data. A One-Sample *T*-Test (after Wilcoxon) was used. A significance was calculated for NIT-treatment and IT-treatment (value of *p* = 0.0039*).

#### 3.2.2. Tumor-necrosis-factor-alpha (Tnfα)

TNF-α originates from the pro-inflammatory cytokine family. Figure 7 illustrates the mean fold expression for NIT versus IT. Control and two different temperature datasets at 37°C and 32°C were used to find out differences in the expression. Every data box for Tnfα experimental setup represents the mean value of four replicates (n = 4) and three repetitions (n = 3). Comparing 37°C versus 32°C in NIT, the median of MFU was at around 10^2^ - 10^3^ in the control, and at 37°C compared to 32°C the median leveled at around 10^2^. The IT median level for both 37°C and 32°C was 10^3^. IT leads to an increased median at 10^3^ of MFU and a smaller range of the standard error of mean (SEM) at 32°C. Due to the ±SEM for both treatments (NIT vs. IT), there was a significance found and calculated in a further One-Sample-Wilcoxon-Test (special type for t-Test for non-Gaussian distributed data). For NIT as well as for IT, the same significance was calculated (value of *p* = 0.0039**) (Figure 7).

**Figure 8:**
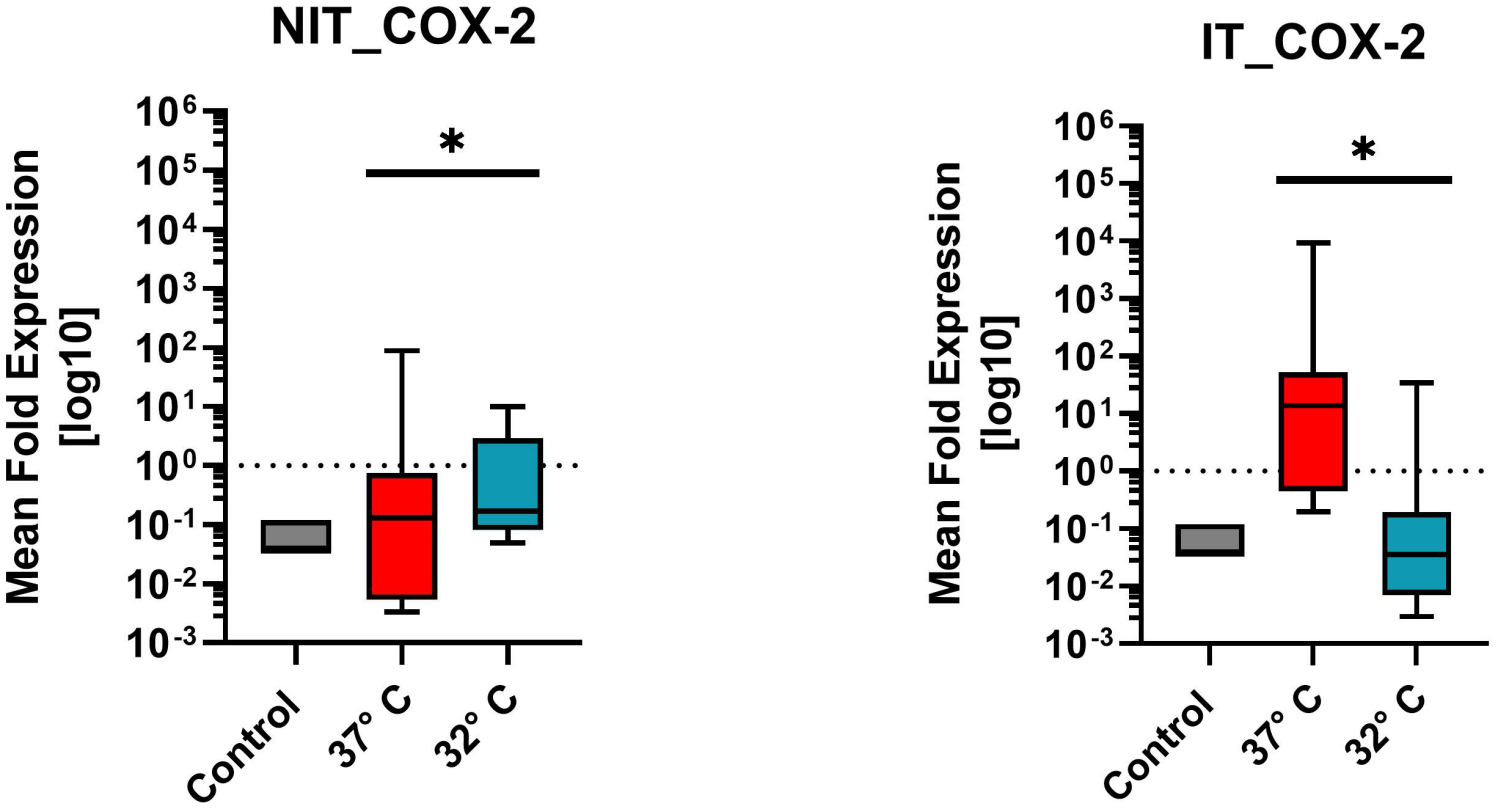
Comparison of mean fold expression of pro-inflammatory enzyme Cox2 for NIT treatment as well as IT treatment. Control and two different temperature datasets at 37°C and 32°C were used to find out differences in the expression. A difference of the median can be recognized for IT at 37°C by the factor of “100.” For IT-treatment and for NIT-treatment there was a significant difference of value of *p* = 0.0039*.

**Figure 9:**
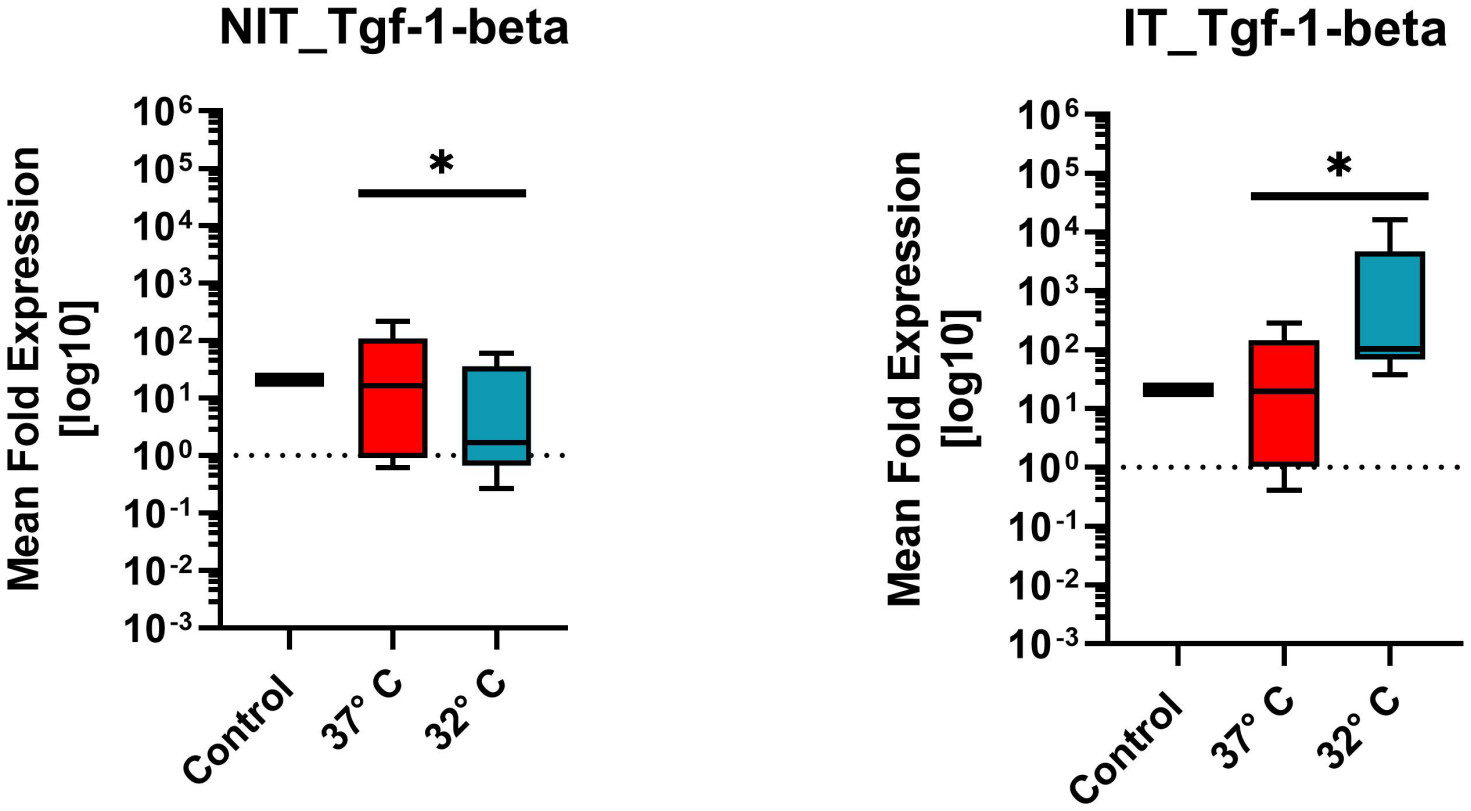
Comparison of mean expression fold change of wound healing growth factor TGF1β for NIT treatment as well as IT. Control and two different temperature datasets at 37°C and 32°C were used to find out differences in the expression. The data of all four markers are not evenly distributed and therefore a one sample *T*-Test (after Wilcoxon) was used. For both treatments (IT and NIT) a calculated significance can be shown (value of *p* = 0.0039*).

#### 3.2.3. Cyclooxigenase-2 (Cox2)

Figure 8 illustrates the mean fold expression of pro-inflammatory enzyme Cox2 for NIT versus IT. Control and two different temperature datasets at 37°C and 32°C were used to find out differences in the expression. Every data column represent the mean value of four replicates (*n* = 4) as well as three repetitions (*n* = 3). The median of MFU was 0.1 in the control group, comparing 37°C vs. 32°C, in NIT, the median of MFU was around 0.1 in both cases. The IT median level for 37°C was at 10^1^ and for 32°C at 10^−1^. A difference of the median can be recognized for IT by the factor of “100″. Due to the high ±SEM for NIT, a significance was calculated in the One-Sample-Wilcoxon-Test showed a high significance (value of *p* = 0.0039 **) for NIT and IT as well.

#### 3.2.4. Transforming-growth-factor-1-beta (Tgf1β)

Tgf1β belongs to the wound healing growth factor group. The mean expression fold change, illustrated in Figure 9 for NIT as well as IT. Control and two different temperature datasets at 37°C and 32°C were used to find out differences in the activation. Every experimental setup represents the mean of four replicates (*n* = 4) and three repetitions (*n* = 3). The control samples showed expression level “15” in NIT comparing 37°C and 32°C, the median of MFU was around 10 at 37°C compared to 32°C on which the median was at 1.1. The IT median leveled for 37°C at around 1.2 * 10^1^ and for 32°C at 10^2^. A difference of the median could be observed in both cases by the factor of “10.”

Due to the ±SEM for NIT treatment, there was a significance calculated in the One-Sample Wilcoxon-Test showed a higher significance (value of *p* = 0.0039 **) for NIT as well as for IT (Figure 9).

## 4. Discussion

In the present work, these pathophysiologic mechanisms were reproduced in a whole organ cochlea culture of 10 day old C57BL6/J mice and evaluated in therapeutic hypothermic conditions. The application of therapeutic hypothermia (32°C culture temperature) (Han et al., 2012) resulted in a downregulation of the pro-apoptotic proteins BAX, CC3 as well as an upregulation of the anti-apoptotic BCL-2 (Figures 4A–F). Hypothermia further decreased the inflammatory cytokines TNFα, Il1ß and COX2 (Figures 6–8). In contrast to that, the expression of the TGF-β1 was increased (Figure 9).

The utilized whole organ cochlea culture method has been first described Hahn et al. in 2008 and further optimized to 7 day old mice (Hahn et al., 2008; Arnold et al., 2010). The applied whole organ cochlea culture technique has been extended to a period of 10 days. A possible limitation of the presented data results from timepoint of preparation of the inner ears till the application of the rotating culture conditions. In this timespan the cochlea were prepared–preparation, insertion—in cooled conditions. Therefore, the temperature effect on the culture starts delayed after acclimatization of the 10 ml culture vessel to the culturing conditions in the incubator. As further limitation of the presented culture experiment, the 24 h culturing time needs to be addressed. Whereas, the apoptosis reaction can be expected in the first 24 h of the insertion trauma to reach the maximum, the additionally tested fibrosis reactions can be expected to reach the maximum in 72 h as described by Bas et al. (2012). A further discussable point is the application of the neurotrophic factors NT-3 and BDNF. Obtaining an informative result from the whole organ cochlea culture model depends on the number of surviving cells. The application of neurotrophic factors as described above to neuronal cultures—like the used whole organ cochlea culture—has been shown to promote cell survival (Schmidbauer et al., 2023). Therefore, the influence of these factors on the reported results need to be discussed. The resulting bias is limited from our point of view because all included groups—Normothermia, Hypothermia, non-insertion trauma, insertion trauma—have been treated with equal concentrations. Furthermore, the neurotrophic factors rather antagonize the measured significant result of a pro apoptotic stimulation. The compromise in the culturing time and neurotrophin application supported significantly the additional focus on the optimal structure preservation as shown in Figure 1. The semi-thin sections of Figure 1 show a good structure preservation at the entire cochlea after a culturing period of 24 h. The integrity of the inner ear structures is further underlined by the selective Myosin VIIa staining of the hair cells. Myosin VIIa is a hair cell marker indicating hair cell survival (Wu et al., 2020). The integrity of the hair cells is indicated with a selective staining in Figures 2C,D in the organ of Corti marking the IHC and OHC. The integrity of the neuronal structures were documented with a selective β-III-Tubulin labeling (Renauld et al., 2015). In Figures 3C,D the neuronal fibers connecting the organ of Corti and the spiral ligament are visualized. Furthermore, the spiral ganglion Cells in Figures 3E,F are typically labeled. The high structural preservation and the typical reactivity of the culture for Myosin VIIa and β-III-Tubulin proof the quality of the whole organ cochlea culture. The good structural preservation of the included cochleae after a 24 h period of culturing confirm the vitality of the examined inner ears. The question of vitality is further underlined by the positive Myosin VIIa staining, which is associated with survival of the by far most delicate hair cells (Wu et al., 2020). The integrity of the further tested neuronal structures is visualized by the positive β-III-Tubulin labeling (Renauld et al., 2015).

The examination of the apoptosis cascade reveals a pro-apoptotic stimulus at 37°C examining the spiral ganglion cells. This can be observed in the NIT and IT group with a higher amplitude in the implanted collective as shown in Figures 5A,B,D,E and Table 1. This observation is based on an upregulation of BAX and CC3 and a downregulation of the anti–apoptotic BCL 2 reaction in the 37°C results as visualized in Figures 4A,F. The quantitative evaluation of the included specimen (*n* = 12) further supports this observation (Chao and Korsmeyer, 1998). The apoptotic activity in the NIT group was expected, because the preparation of the cochlea has to be considered a cochlea trauma including an axotomy situation. Nevertheless the spiking of the IT group is obvious induced by the electrode insertion. The expression pattern in the 32°C group significantly changes in favor of an anti-apoptotic situation. Compared to the 37°C treated cultures a lower signal intensity is noted for BAX and CC3 (Figures 4B,D, 5A,B,D,E), whereas a higher intensity is shown in the BCL2 slides (Figures 4F, 5C,F). The quantitative evaluation in the spiral ganglion cells further support this. The compared changes show significant differences between the 37°C and the 32°C group in the IT and NT group. Nevertheless, the overall percentage of positive cells was obvious higher in the IT group compared to the NIT group. This constant difference has to be accounted to the insertion trauma set in the experiment (Table 1). For CC3, Bax and Bcl2, calculated significances for IT are in the same range (value of *p* < 0.0001). For IT, calculated value of p for CC3 was exact at 0.0001 (value of *p* = 0.0001). For Bax and Bcl2 was a lower significance was calculated (value of *p <* 0.0001). The expression pattern at 32°C confirms an anti-apoptotic effect of therapeutic hypothermia in the IT and NIT group with a higher effect in the IT group (Chao and Korsmeyer, 1998). Transferring these *in vitro* observations into a clinical situation an otoprotective effect of therapeutic hypothermia on the electrode insertion trauma by downregulation of the apoptosis cascade can be posted.

In addition to the apoptosis cascade the Il1ß was further evaluated. The rtPCR analysis for Il1β revealed an upregulation of the Il1ß in the IT group at 37°C. This effect was significantly lower in the 32°C group suggesting therapeutic effect of the applied hypothermia on the Il1ß pathomechanism (Figure 6). Il1ß is a well-known pro-inflammatory cytokine involved in a plethora of various complex sequences of the immune system inducing the expression of various target genes, such as Interleukin-6 (Il6), Interleukin-8 (Il8) and Cox2 (Weber et al., 2010). The upregulation of Il-1β in an experimental electrode insertion trauma in a whole organ cochlea culture setting has been shown by Bas et al. (2012). Therefore, this observation is conclusive with the literature. The decreasing effect of therapeutic hypothermia on the Il1β expression has been prior shown in various clinical setting, like cerebral infarction (Li et al., 2022) or renal reperfusion injury (Schleef et al., 2022). The reduction of the Il1β levels in cochleae with an electrode IT through therapeutic hypothermia is a new finding.

As a strong pro-inflammatory cytokine Il1β is known to induce the pro-inflammatory enzymes like COX2 (Weber et al., 2010). The evaluation of the COX2 expression in the electrode IT group revealed an increase of COX2 in the normothermic group. A finding prior published by Bas et al. (2012). In the hypothermic group, the COX2 levels were significantly lower (Figure 8). The COX2 expression has been associated with inflammation and the formation of ROS. The COX2 level relates proportionally to the cytotoxic and neurotoxic damage of the inflammatory process (Nogawa et al., 1997). The capacity of mild therapeutic hypothermia of the Il1β and COX2 axis further supports the hypothesis of an otoprotective effect of mild therapeutic hypothermia in an electrode insertion trauma.

As further fundamental factor in acute inflammation, the TNFα expression has been tested. TNFα is known as a pro-inflammatory cytokine mediating pleiotropic effects like apoptosis, cell proliferation, and cytokine production (Horiuchi et al., 2010). The expression pattern in this analysis is similar to the above described findings. In the electrode IT group, an elevation of the TNFα mean fold expression can be posted for the 37°C group (Figure 7). At 32°C, no elevation could be observed. This expression pattern is conclusive with the rest of the above described finding in reference to the Il1β expression and activation of the apoptosis cascade (Frejo and Lopez-Escamez, 2022). In an experimental electrode IT study, the elevation of TNFα has been prior described (Bas et al., 2012). This reductive effect of therapeutic hypothermia is a novelty.

Furthermore, the bearing of TGFβ1 in the experimental electrode IT has been evaluated. TGFβ1 is a highly pleiotropic cytokine with multiple functions including anti-inflammatory capacity, fibrosis and angiogenesis (Prud’homme, 2007). As presented in Figure 9 the application of therapeutic hypothermia leads to a significant upregulation of TGFβ1 in comparison to the normothermic group. Comparing this finding with the prior describe electrode IT pathomechanism, the results were comparable with a low expression of TGFβ1 in the untreated group and elevation in the otoprotective group. To solidly interpret the elevation of TGFβ1 in the hypothermic group, further experiments are necessary. At the current stage, both an anti-inflammatory stimulus and a fibrotic stimulus could be possible (Prud’homme, 2007). With the current knowledge, an involvement of the TGFβ1 cytokine cascade in the effect of therapeutic hypothermia in the electrode IT pathophysiology could be shown.

These conclusive pathophysiologic findings have been achieved in an experimental set up with a hypothermic exposure over 24 h. This application time cannot be directly transferred into human use. The optimal ratio of hypothermia application time in relation the achieved functional protection needs further investigation. With an *in vivo* hypothermia application of 40 min in a rodent CI insertion model Tamames et al. (2016) provided the first clue of a executable timespan (Tamames et al., 2016). In addition, the cadaver study by Bader et al. revealed an effective local hypothermia application to the whole human cochlea utilizing a room temperature rinse of the cochlea implantation situs (Bader et al., 2020). Respecting these two milestones applying local hypothermia with a post insertion rinse for 40 min after insertion seems feasible. Nevertheless, the issue of the limited application time of local hypothermia needs to be considered. The application of local hypothermia is performed in general anesthesia, limiting it to a short period within the procedure. Other application forms of otoprotection, like drug eluting electrodes, effect the equal pathomechnisms as discussed above for a longer time period. (Eshraghi et al., 2019) If a short immediate otoprotective impulse, as provided by local hypothermia, results in a sufficient effect further studies are necessary to confirm this hypothesis.

The presented pathophysiologic effect of hypothermia on the electrode insertion trauma further supports the usability of this otoprotective option. As to the current stand of the literature, hypothermia, on the one hand, protectively effects the pathophysiology of the electrode insertion trauma. On the other hand, the previously published study by Bader et al. provides a feasible intraoperative application form, utilizing a standard established intraoperative rinsing as cooling source. The intraoperative irrigation was applied over the posterior tympanotomy into the middle ear. With a room temperature rinse (22° Celsius) the intracochlear temperature was shifted into hypothermic conditions through the entire inner ear (Bader et al., 2020). With these two milestones established, the next step is a trail in a clinical setting using intraoperative saline rinsing for 40 min after the insertion trauma.

Summarizing elaborated the results, it can be posted that the electrode IT leads to a pro-inflammatory cytokine (TNFα, Il1β) and enzyme (COX2) expression as well as a pro-apoptotic stimulus with elevated BAX and CC3 activation in the cochlea. This reaction can be reversed with the application of therapeutic hypothermia resulting in significant lower pro-inflammatory cytokine (TNFα, Il1β) and enzyme (COX2) expression as well as an anti-apoptotic immunohistochemical expression pattern with an upregulated BCL2 and downregulated BAX and CC3 expression. These results suggest an otoprotective effect of therapeutic hypothermia in regard to the early inflammatory response stage of electrode IT.

## Data availability statement

The original contributions presented in the study are included in the article/Supplementary material, further inquiries can be directed to the corresponding author.

## Ethics statement

Ethical review and approval was not required for the animal study because Extraction of tissue from euthanized animals conforms with the Austrian Federal act on Experiments of Living Animals (Tierversuchsgesetz 2012 – TVG 2012, §2) based on the EU Directive 2010/63/EU.

## Author contributions

JS, WB, and AS-F: idea, conceptualization, methodology, writing, and supervision. WB and JD: formal analysis, investigation, and data curation. TG, DD, and RG: validation and reviewing and editing. All authors contributed to the article and approved the submitted version.

## Funding

This study received funding from by MEDEL Medical Electronics, Headquarters Fürstenweg 77a, 6020 Innsbruck, Austria. In addition, this work was also supported by Austrian Science Fund (FWF) Grant No. I4147-B.

## Conflict of interest

The authors declare that the research was supported by MEDEL science grant, Medical Electronics, Headquarters Fürstenweg 77a, 6020 Innsbruck, Austria. The concept and realization of the study was done alone by the authors.

## Publisher’s note

All claims expressed in this article are solely those of the authors and do not necessarily represent those of their affiliated organizations, or those of the publisher, the editors and the reviewers. Any product that may be evaluated in this article, or claim that may be made by its manufacturer, is not guaranteed or endorsed by the publisher.

## Figures and Tables

**Table:** 

C57BL6/J	C57 Black 6 Mice
°C	Degree Celsius
BAK	B-cell-Lymphoma-2-Homologous-Antagonist/Killer protein/gene
BAX/Bax	B-Cell-Lymphoma-2-Associated-X protein/gene
BCL2/Bcl2	B-Cell-Lymphoma-2 protein/gene
BDNF	Brain Derived Neurotrophic Factor protein
CC3/Cc3	Cleaved-Caspase-3 protein/gene
cDNA	Complementary deoxyribonucleic acid
control	Control group
COX2/Cox2	Cyclooxygenase-2-protein
CSV	Comma-Separate-Values (data format)
CTGF	Connective-Tissue-Growth-Factor protein
Ct-value	Cycle threshold value
DAPI	4′,6-diamidino-2-phenylindole (staining)
dNTPs	Deoxynucleotide triphosphates
EDTA	Ethylenediaminetetraacetic acid
FITC	Fluorescein isothiocyanate
HBSS	Hank’s Balanced Salt Solution
ICH	Inner hair cell
IL1β/Il1 β	Interleukin-1-Beta protein/gene
IL-6	Interleukin 6 protein
IL-8	Interleukin 8 protein
iNOS	Inducible Nitric Oxide Synthetase
IT	Insertion trauma
MFU	mean fold units
MgCl^2^	Magnesium chloride
NCBI	National Centre for Biotechnology Information
NIT	Without insertion trauma
NT-3	Neurotrophin-3
OHC	Outer hair cell
PBS	Phosphate-buffered saline
PCR	Polymerase chain reaction
PFA	Paraformaldehyde
qPCR	Quantitative polymerase chain reaction
RCCS	Rotary Cell Culture System
RNA	Ribonucleic acid
ROS	Reactive oxygen species
rt-PCR	Real-time quantitative polymerase chain reaction
SEM	standard error of mean
TGFβ1/Tgfβ1	Transforming-Growth-Factor-Beta-1 protein/gene
TGFβ3/Tgfβ3	Transforming-Growth-Factor-Beta-3 protein/gene
TNFα/Tnfα	Tumor-Necrosis-Factor-Alpha protein/gene
