# Supplementary material for: The effects of mild hypothermia on the electrode insertion trauma in a murine whole organ cochlea culture
Source: Front Neurosci. 2023 Apr 13;17:1112243. doi: 10.3389/fnins.2023.1112243 (PMC10133490; doi:10.3389/fnins.2023.1112243)
Supplement: Supplementary file 1 [file Data_Sheet_1.docx]

Supplementary Material

The effects of mild hypothermia on the electrode insertion trauma in a murine whole organ cochlea culture

Joachim Schmutzhard^1*†^, Werner Bader^1†^, Timo Gottfried^1^, Daniel Dejaco^1^, …, Joszef Dudas^1^, Annelies Schrott-Fischer^1^

^1^Department for Otorhinolaryngology, Head and Neck Surgery, Medical University of Innsbruck, Innsbruck, Austria

**†**These authors equally contributed to this work.

*** Correspondence:**Assoz. Prof. Priv.-Doz. Dr. med. univ. Joachim Schmutzhard PhD
joachim.schmutzhard@i-med.ac.at

# Supplementary table A: Composition of Rotary Cell Culture Media

| **Constituent** | **Final**  **Concentration** | **Vol. 12 ml**  **(one vessel)** | **Vol. 50 ml**  **(four vessels)** |
| --- | --- | --- | --- |
| BDNF | 10 ng/ml | 120 ng | 500 ng |
| NT-3 | 5 ng/ml | 60 ng | 250 ng |
| Neurobasal Media (Gibco, 21103-049) |  | 9,35 ml | 46,75 ml |
| L-Glutamine (200 mM) | 5 mM | 250 µl | 1,25 ml |
| HEPES-Buffer (1 M, PAA, S11-001) | 10 mM | 100 µl | 500 µl |
| Penicillin G (20000 units) | 100 units/ml | 100 µl | 500 µl |
| B27 Supplement (Invitrogen, 17504-044 50x stock) | 1x | 200 µl | 1,00 ml |
| NaOH (for pH adjustment to 7,4) | 1 M | 30 µl | 150 µl |

# Supplementary table B: Forward- and Reverse Primer for Il-1-ß, Tnf-alpha, Tgf-1-beta and Cox-2

|  | **Forward (5‘ – 3‘)** | **Reverse (5‘ – 3‘)** | **Sequence length  [base pairs]** |
| --- | --- | --- | --- |
| Interleukin-1-beta | ACTCATTGTGGCTGTGGAGA | TTGTTCATCTCGGAGCCTGT | 199  base pairs |
| Tnf-alpha | ATTCTGGAGAGCAGAGCGAG | GATCTTGTGGGCCTCCTTCT | 221  base pairs |
| Tgf-1-beta | ATGAAAAGGCTCCCGAGGAA | TGTGGCTCCCCTTCAGTTAG | 212  base pairs |
| Cox-2 | GACAAATCAACAACCCCGT | TGGCAGAACGACTCGGTTAG | 174 base pairs |

# Supplementary table C: Components for a 22 µl reaction master mix

| **Components** | **Volumn [in µl]** |
| --- | --- |
| Destilled Water | 7,2 µl |
| Forward Primer | 0,9 µl |
| Reverse Primer | 0,9 µl |
| 2x SybrGreen | 11,0 µl |
| cDNA | 2,0 µl |
| **Total** | **22,0 µl** |

# Supplementary table D: Composition of one PCR-Cycle for quantitative PCR

| **Temperature** | **Cycle stage** |
| --- | --- |
| 95° C for 5 sec. | Denaturation |
| 60° C for 10 sec. | Annealing |
| 72° C for 5 sec. | Elongation |

**PCR-Cycle**; for qPCR experiments a thermocycler (Bio-Rad MyiQ realtime instrument, by Bio-Rad) was used according to following technical settings (95° C for cDNA; 3 min. and 40 Cycles).

# Supplementary protocol: Cryo-Embedding

**Day 1:**

Decalcified specimen are rinsed using PBS (1x). Further a 10 % Sucrose solution [in PBS (1x)] was used and put on the lab shaker for 30 minutes. Followed by using a 15 % Sucrose solution [in PBS (1x)] for 30 minutes on the lab shaker again. Subsequently, specimens are stored over night (o/n) on 4° C in the fridge.

**Day 2:**

A mixture of 15 % sucrose in PBS (1x) and OCT (by Scigen) in relation 1:1 was used at 4° C on the lab shaker.

**Day 3:**

Specimens were slightly vacuumed in 100 % OCT (by Scigen) to get rid of air bubbles from the tissue and put over night (o/n) at 4° C on the lab shaker.

**Day 4:**

Small pyramid-shape tins were filled with pure OCT (by Scigen) (100 %) and vacuumed for one hour. Specimens were then transferred and oriented by using a microscope needle. Cochleae were then frozen on mud containing CO_2_ and Ethanol (EtOH) and stored at - 20° C (short term storage) or at - 80° C (long term storage).

**PROTOCOL FROZEN 402 for the special use of Cryo-Sections**

Protocol # 402 : FISH_Cryo (02.12.2021) Procedure: Research FISH Discovery Staining Module Step No Procedure Step

1 ***** Start Timed Steps *****

2 ***** Select Reaction Buffer *****

3 Rinse Slide +

4 Adjust Slide Volume

5 Apply Coverslip

6 Warmup Slide to 37 Deg C, and Incubate for 2 Minutes

7 ***** Select EZ Prep *****

8 ***** Select SSC Wash *****

9 ***** Select Reaction Buffer *****

10 ***** Select EZ Prep *****

11 Disable Slide Heater

12 Rinse Slide +

13 Adjust Slide Volume

14 ***** Select SSC Wash *****

15 Warmup Slide to 37 Deg C, and Incubate for 2 Minutes

16 Rinse Slide +

17 Adjust Slide Volume

18 Apply Coverslip

19 Warmup Slide to 37 Deg C, and Incubate for 2 Minutes

20 Disable Slide Heater

21 ***** Select Reaction Buffer *****

22 Warmup Slide to 37 Deg C, and Incubate for 2 Minutes

23 Rinse Slide +

24 Adjust Slide Volume

25 Apply Coverslip

26 ***** Mixers Off *****

27 ***** Wait For Button ( Hold 1st Ab titration ) *****

28 ***** Mixers On *****

29 Rinse Slide +

30 Adjust Slide Volume

31 Apply Coverslip

32 ***** Hand Apply ( Primary Antibody ) *****

33 Incubate for [60 Minutes] ( Primary Antibody )

34 Rinse Slide +

35 Adjust Slide Volume

36 Apply Coverslip

37 ***** Mixers Off *****

38 ***** Wait For Button ( Hold 2nd Ab titration ) *****

39 ***** Mixers On *****

40 Rinse Slide +

41 Adjust Slide Volume

42 Apply Coverslip

43 ***** Hand Apply ( Secondary Antibody ), and Incubate for [60 Minutes] *****

44 Rinse Slide +

45 Apply Coverslip ENT Research Lab, Anichstr. 35 A-6020 Innsbruck * one drop is one reagent dispense Printed 05.05.2022 15:45:37 Page 1 of 2 NexES v10.6 Protocol # 402 : FISH_Cryo (02.12.2021) Procedure: Research FISH Discovery Staining Module Step No Procedure Step

46 ***** Select EZ Prep *****

47 ***** Select Reaction Buffer *****

48 Rinse Slide +

49 Apply Coverslip

50 Disable Slide Heater

51 ***** Mixers Off *****

52 ***** Wait For Button ( Hold Counterstain/Cleaning ) *****

53 ***** Mixers On *****

54 ***** Select EZ Prep *****

55 ***** Select Reaction Buffer *****

56 Rinse Slide +

57 Rinse Slide +
